# Pandemic (H1N1) 2009 Surveillance in Marginalized Populations, Tijuana, Mexico

**DOI:** 10.3201/eid1608.100196

**Published:** 2010-08

**Authors:** Timothy C. Rodwell, Angela M. Robertson, Norma Aguirre, Alicia Vera, Christy M. Anderson, Remedios Lozada, Lwbba Chait, Robert T. Schooley, Xing-quan Zhang, Steffanie A. Strathdee

**Affiliations:** University of California, San Diego, California, USA (T.C. Rodwell, A.M. Robertson, N. Aguirre, A. Vera, C.M. Anderson, L. Chait, R.T. Schooley, X.-Q. Zhang, S.A. Strathdee); Patronato Pro-COMUSIDA, Tijuana, México (R. Lozada)

**Keywords:** Viruses, influenza, respiratory infections, pandemic (H1N1) 2009, surveillance, at-risk populations, Mexico, dispatch

## Abstract

To detect early cases of pandemic (H1N1) 2009 infection, in 2009 we surveyed 303 persons from marginalized populations of drug users, sex workers, and homeless persons in Tijuana, Mexico. Six confirmed cases of pandemic (H1N1) 2009 were detected, and the use of rapid, mobile influenza testing was demonstrated.

The first declared influenza pandemic in 40 years likely originated in March 2009 in La Gloria, Veracruz, a small rural town in southern Mexico (*1*). The virus responsible for the outbreak, identified as a novel influenza A (H1N1) virus, now referred to as pandemic (H1N1) 2009, spread quickly. By the end of 2009, ≈70,000 cases were confirmed and 944 deaths were recorded in Mexico (*2*), and >600,000 cases were reported worldwide (*3*).

When pandemic (H1N1) 2009 first emerged there was concern that Mexico–US border cities, which have served as corridors for binational transmission of infectious diseases (*4*), might become overwhelmed by the disease before national surveillance resources could be mobilized. Of particular concern was the fact that traditional hospital-based disease surveillance would miss the emergence of pandemic (H1N1) 2009 in the dense, highly mobile (*5*), marginalized border populations. Furthermore, the poorest and most underserved border populations, composed of the homeless, commercial sex workers, and those with alcohol and drug abuse problems, have disproportionately high levels of diseases such as HIV (*6*) and tuberculosis (*7*), illnesses suspected of increasing vulnerability to acquiring and dying of pandemic (H1N1) 2009 (*8**,**9*). We report the results of an enhanced surveillance effort to detect early pandemic (H1N1) 2009 cases and assess perceptions of the pandemic and risk factors associated with acquiring the disease in marginalized populations in Tijuana, Mexico.

## The Study

This surveillance project was a collaboration between Mexican public health officials, nongovernment organizations, and the University of California, San Diego (UCSD). The project was approved by the institutional review boards of Tijuana General Hospital and UCSD.

Our binational research team of US and Mexican researchers has been conducting prospective studies of populations at high risk for infectious diseases in the most vulnerable communities of Tijuana and Ciudad Juarez, Mexico, since 2004 (*10**,**11*). More than 2,500 drug users, sex workers, and homeless persons have been recruited to participate in those studies. From May 1 through November 20, 2009, we conducted active and passive influenza surveillance within our existing marginalized study populations in Tijuana. We used mobile outreach units and community clinics in the city’s Zona Roja (red light zone) as magnet sites (Figure).

**Figure:**
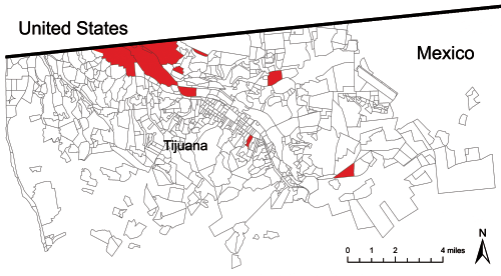
Red shaded areas indicate *colonias* of Tijuana, Mexico where pandemic (H1N1) 2009 virus screening took place, May 1–November 20, 2009.

Active surveillance included contacting all known current and previous study participants, parking mobile health units in study zones (Figure), and having outreach workers refer persons who reported feeling unwell for any reason. Passive recruitment, conducted by word-of-mouth advertising, was open to all persons in communities served by our mobile and fixed-site clinics.

Persons seen by the surveillance team had their oral temperatures recorded and completed brief surveys documenting demographics, influenza risk factors, and clinical symptoms. Persons meeting inclusion criteria for influenza-like illness based on early Centers for Disease Control and Prevention guidelines (*12*) were considered pandemic (H1N1) 2009 suspected case-patients. Inclusion criteria were a subjective or objective fever (oral temperature >100°F [37.8°C]) and 1 of the following signs or symptoms: cough, sore throat, severe headache, diarrhea, vomiting, or severe muscle/body aches and fatigue.

Persons with influenza-like illness completed an in-depth survey and underwent rapid influenza testing. Participants were compensated $5 US. In-depth surveys included questions on symptom duration, treatment choices, and pandemic (H1N1) 2009 knowledge.

Enrolled pandemic (H1N1) 2009 suspected case-patients provided a nasopharyngeal swab for rapid influenza testing with the BinaxNOW assay (Inverness Medical, Waltham, MA, USA). Suspected case-patients with positive rapid test results for type A influenza were considered probable pandemic (H1N1) 2009 case-patients and provided another nasopharyngeal swab for influenza A strain confirmation and characterization. Persons with positive test results were transported to Tijuana General Hospital for evaluation and treatment. Suspected case-patients with negative rapid influenza test results were not evaluated further.

Nasal swab specimens were cultured on MDCK cells within 48 hours of field collection. Positive cultures were detected by cytopathic effect and by hemabsorbtion. Viral isolates were characterized by hemagglutinin and neuraminidase type by using immunofluorescent staining (ViroStat, Portland, ME, USA and eEnzyme, Gaithersburg, MD, USA) and confirmed to be pandemic (H1N1) 2009 by real-time PCR (*13*).

From May 1 through November 20, 2009, a total of 303 persons were screened. Forty-three (15%) met inclusion criteria as pandemic (H1N1) 2009 suspected case-patients and were tested with the rapid influenza test. Six suspected case-patients (14%) had rapid test results positive for influenza A, and all 6 were confirmed to have influenza A pandemic (H1N1) 2009 virus. One patient had a rapid test result positive for influenza B. Pandemic (H1N1) 2009 cases were not related and were detected at a rate of ≈1/ month during July–November.

Median age of screened persons was 35 years (interquartile range 29–43 years), and 62% were men (Table). Median oral temperature of patients with confirmed pandemic (H1N1) 2009 was 37.9°C (IRQ 37–38.3). Six (86%) of 7 patients with influenza reported a cough and 5 (71%) reported sore throat or severe joint/muscle ache. Less than 10% of screened persons not identified as influenza suspected case-patients had these symptoms. No patients with confirmed influenza reported vomiting or diarrhea.

**Table:** Univariate comparisons of 303 persons screened for pandemic (H1N1) 2009 virus in Tijuana, Mexico, May 1–November 20, 2009*

Variable	Recruits not meeting inclusion criteria, n = 260	Suspected case-patients negative for influenza A/B,† n = 36	Suspected case-patients positive for influenza A/B,† n = 7
Age, y	35 (29–43)	34.5 (31.5–42.5)	28 (6–48)
Male sex	162 (63.3)	20 (55.6)	3 (42.9)
Heard about “swine flu” epidemic before screening	228 (87.7)	30 (90.9)	6 (85.7)
Subjective fever	14 (5.4)	13 (37.1)	7 (100)
Objective fever, oral temperature, °C	36.5 (36.2–36.8)	37.1 (36.6–37.5)	37.9 (37–38.3)
Cough	27 (10.4)	15 (42.9)	6 (85.7)
Sore throat	16 (6.2)	18 (51.4)	5 (71.4)
Severe headache	40 (15.4)	23 (65.7)	4 (57.1)
Joint aches	16 (6.2)	16 (47.1)	5 (71.4)
Muscle aches	18 (6.9)	18 (52.9)	5 (71.4)
Severe fatigue	23 (8.9)	17 (48.6)	5 (71.4)
Diarrhea	12 (4.6)	9 (25.7)	0
Vomiting	10 (3.9)	5 (14.3)	0
Traveled outside Tijuana since symptoms started	10 (3.9)	7 (20.0)	3 (42.9)
Contact with possible “swine flu” case in past 2 wks	6 (2.3)	11 (31.4)	4 (57.1)
Ever received influenza vaccination	13 (5.0)	5 (14.3)	6 (85.7)
Heard about “swine flu” from friends	74 (32.5)	11 (37.9)	5 (83.3)
Heard about “swine flu” from family	22 (9.6)	7 (24.1)	4 (66.7)
Heard about “swine flu” from co-workers	14 (6.1)	7 (24.1)	1 (16.7)
Heard about “swine flu” from newspaper	33 (14.5)	3 (10.3)	2 (33.3)
Heard about “swine flu” from radio	80 (35.1)	12 (41.4)	6 (100.0)
Heard about “swine flu” from television	182 (79.8)	23 (79.3)	6 (100.0)
>3 alcoholic drinks/d‡		5 (14.7)	0
Injection drug user‡		16 (45.7)	0
In jail 2 weeks before onset of symptoms‡		2 (5.7)	0
Would be vaccinated for “swine flu” ‡		36 (100.0)	7 (100.0)
Prefer to be vaccinated at hospital‡		25 (71.4)	5 (71.4)
Prefer to be vaccinated at clinic‡		1 (2.9)	0
Prefer to be vaccinated at PrevenCasa/Casita‡		5 (14.3)	3 (42.9)
Prefer to be vaccinated at mobile clinic ‡		0	0

*Values are no. (%) except for age and objective fever, which are median (interquartile range). †By rapid influenza testing. ‡Only pandemic (H1N1) 2009 suspected case-patients (n = 43) were asked these questions.

All patients with confirmed pandemic (H1N1) 2009 received antiviral drug treatment within 24 hours of their positive rapid influenza test result; no pandemic (H1N1) 2009 related deaths were reported by completion of the study. One person with a negative rapid test result who was severely ill during the survey died within days of being referred to Tijuana General Hospital but was diagnosed with end-stage tuberculosis after death, not influenza.

Awareness was high; 88% of screened persons (n = 264/300) who answered the question about prior knowledge reported hearing about the pandemic (H1N1) 2009 epidemic in Mexico before the time of screening. More than 80% heard about the epidemic from television broadcasts. All pandemic (H1N1) 2009 suspected case-patients (n = 43) reported that they would get a pandemic (H1N1) 2009 vaccine if available; most suspected case-patients (71%) preferred to be vaccinated in a hospital.

## Conclusions

Of ≈1,200 persons and their families who were contacted directly during the course of this survey, 303 self-identified as “feeling unwell” during May 1–November 20, 2009. Six persons had confirmed pandemic (H1N1) 2009 virus, but given that the rapid influenza test used for screening in this study has only moderate sensitivity (*14*), this is likely an underestimate of cases. Confirmed cases represented ≈0.7% of the 826 pandemic (H1N1) 2009 cases recorded in Tijuana during April–November 2009 (*15*), suggesting that isolation of these populations from the general population might have been protective.

All case-patients were treated with antiviral medication within 24 hours of a rapid influenza A positive test result, but access to antiviral drugs was through nontraditional means requiring many levels of negotiation by study staff and medical professionals in Tijuana that would not have been practical if more cases had been detected. The antiviral medication, oseltamivir, although plentiful in the United States, was only available in limited quantities in Tijuana, indicating that there are major hurdles to overcome in binational distribution of medical resources in an early pandemic setting.

Although rapid influenza tests are relatively insensitive, especially for pandemic (H1N1) 2009 (*14*), these tests can identify persons for PCR confirmation in an efficient and cost-effective fashion for early detection of cases. If pandemic (H1N1) 2009 were to develop more extensively in this population, changing inclusion criteria to objective fever (>100°F [37.8°C]) plus sore throat and/or cough would likely be effective for identifying pandemic (H1N1) 2009 cases with greater precision. Despite the limited health, financial resources, and unstable housing in our study population, it appears that television broadcasts were effective in distributing early messages about pandemic (H1N1) 2009. This enhanced surveillance survey, planned and executed safely and efficiently within 4–6 weeks of the initial pandemic (H1N1) 2009 cases in Mexico, demonstrated the potential of binational academic/public health collaborations to respond to emerging health threats in Mexico/US border regions in real time.
